# African Primary Care Research: Qualitative data analysis and writing results

**DOI:** 10.4102/phcfm.v6i1.640

**Published:** 2014-06-06

**Authors:** Langalibalele H. Mabuza, Indiran Govender, Gboyega A. Ogunbanjo, Bob Mash

**Affiliations:** 1 Department of Family Medicine, Medunsa Campus, University of Limpopo, South Africa; 2 Family Medicine and Primary Care, Stellenbosch University, South Africa

## Abstract

This article is part of a series on African primary care research and gives practical guidance on qualitative data analysis and the presentation of qualitative findings. After an overview of qualitative methods and analytical approaches, the article focuses particularly on content analysis, using the framework method as an example. The steps of familiarisation, creating a thematic index, indexing, charting, interpretation and confirmation are described. Key concepts with regard to establishing the quality and trustworthiness of data analysis are described. Finally, an approach to the presentation of qualitative findings is given.

## Abstrait

**Recherche en Soins primaires africains: analyse de données qualitatives et rédaction des résultats**. Cet article fait partie d'une série sur la recherche en soins primaires africains et offre des conseils pratiques pour l'analyse des données qualitatives et la présentation des résultats qualitatifs. Après un survol des méthodes qualitatives et des approches analytiques, l'article se penche particulièrement sur l'analyse du contenu, en utilisant comme exemple la méthode du cadre logique. On y décrit les étapes de familiarisation, la création d'un index thématique, l'indexage, le répertoriage, l'interprétation et la confirmation. Les concepts clés concernant la détermination de la qualité et de la fiabilité de l'analyse des données sont définis. Finalement, on y donne une méthode de présentation des résultats qualitatifs.

## Introduction

This article is part of a series on African primary care research and addresses the topics of qualitative data analysis and writing results. Over the last few decades there have been several improvements in the scientific rigour of qualitative research methods, which had previously been regarded as being less scientific than more established quantitative approaches.^1, 2^

In the discipline of family medicine and primary care, most researchers have been using focus group or in-depth interviews to collect qualitative data. Few researchers engage with participant or non-participant observation or immerse themselves for prolonged periods in other social groups or cultures. Research questions often focus on the need to gain a better understanding of the experiences of people regarding health-related topics or the experiences of patients regarding health services. The need to explore or understand people's perceptions, attitudes, beliefs, concerns, feelings or expectations are often mentioned in the aim and objectives. Whilst the qualitative research field is a broad one (see Table 1), this article will focus mainly on the analysis and reporting of qualitative data derived from interviews. This article complements the other article in this series on qualitative interviewing and methods.

**TABLE 1:** Range of qualitative methodological approaches and analysis.

Type of qualitative research	Description
Ethnography^3^	This refers to the study of culture or cultures shared by a group of people. The investigator immerses him- or herself in the group for a long period of time (a year or more), gradually establishing trust and experience in the social world of the participants.
Netnography (Cyberethnography/virtual ethnography)^4^	This is a method that investigates communities created by a network (e.g. online communities), which are distant from the investigator and dispersed in their nature. Like physical communities, the researcher can study online communities through immersion in the group for an extended period of time.
Ethnomethodology^5^	This focuses on the way that participants construct the social world in which they live (how they ‘create their own reality’) – unlike ethnography which seeks to describe the social world as the participants see it.
Conversation analysis^6^	This focuses on how reality is constructed, rather than what it is. The moment-by-moment conversational interchange of a group of participants is analysed line-by-line to bring about the subtle meaning which the participants may not even be fully aware of.
Narrative analysis^7^	The focus of this analytical method is on understanding the bigger story or ‘narrative’ that people use to make sense of their experiences and events. ‘Narratives must have a point (a “so what?” factor), which often takes the form of a moral message’.
Grounded theory^8, 9^	‘A systematic theory developed inductively, based on observations that are summarized into conceptual categories, re-evaluated in the research setting, and gradually refined and linked to other conceptual categories’.^8^ Often involves a constant comparative analytical method, culminating in the production of a substantive theory.^9^

## Qualitative data

Qualitative data is usually text that has been derived from interviews, observations or already-existing documents. Interviews are usually audio- or video-recorded and must be transcribed verbatim in order to create data that can be analysed further.^3, 4, 5, 6, 7, 8, 9, 10^ The transcript should therefore be a word-by-word account and not paraphrased. Transcripts should include page and line numbers and should have sufficiently large margins to allow for coding.^11, 12^ Before analysing the data the transcript should be checked for accuracy and any mistakes in the transcription corrected against the original recording. If the interview must be translated, then the accuracy of the translation must also be verified. These steps in preparing the data for analysis must be described in the research proposal.

## Approach to qualitative data analysis

Most primary care researchers use content analysis to make sense of their data and most approaches to content analysis follow the same basic stages of familiarisation, coding and formation of categories, interpretation of themes, confirmation of the interpretation and presentation of the results.^13^ Attention must also be given throughout to the trustworthiness of the data and data analysis process, as is discussed below. In order to provide a practical approach to qualitative data analysis, the Framework Method is described in some detail below as one commonly-used example.^14^ In your research proposal, you should outline the steps that you will follow in your analysis.

### Familiarisation

If you are performing your own data collection, this process may start in the field when you collected your data and made field notes. Once the data is available, however, the researcher(s) will make themselves familiar with the qualitative data as a whole by reading the transcripts and observation notes or listening to the tapes. During this process they should take note of key ideas and recurrent themes as they emerge, their own reactions to the data (see ‘Reflexivity’ below) and any remarks on the quality of the data or methodological issues. If there is a large amount of data then it may be necessary to select a representative sample of the data for this stage.^15^

### Development of a thematic index

A list of codes is created based on the familiarisation process mentioned above and organised into categories. Codes and categories should be developed from the data (an inductive process),^16^ but with the aim and objectives of the study in mind, as sometimes the data contains material that is irrelevant. Sometimes the objectives also provide an overall structure for the categories. The final list of codes and categories is called a ‘thematic index’^14, 17^ and an example is given in Box 1 from a study looking at how people experienced the use of anti-retroviral medication. The thematic index can then be tested and refined on a small number of the transcripts or data sources.

BOX 1 Example of a thematic index.

**1.How patients incorporate the taking of ARVs into their lifestyle**

1.1 Use of technology (e.g. cellphone alarm, clock/watch alarm)

1.2 Reminders from family members or treatment supporters

1.3 Intuition (they just feel it is the right time)

1.4 Et cetera

**2.Beliefs and feelings of patients/treatment supporter regarding their ARVs**

2.1 Believe that they make them better (not get AIDS)

2.2 Believe that they increase life expectancy

2.3 Use of ARVs is form of unintended disclosure (that leads to stigma)

2.4 Et cetera

**3. Positive experiences of patients/treatment supporters attending the ARV clinic at the district hospital**

3.1 Caring and supportive staff

3.2 Sharing experiences with other patients

3.3 Regular supply of medication (vs. not regular at local clinic)

3.4 Et cetera

**4. Negative experiences of patients/treatment supporters attending the ARV clinic at the district hospital**

4.1 Uncaring and unsupportive staff

4.2 Blaming or judging person for their problems

4.3 Long queues and waiting times

4.4 Et cetera

ARV, antiretroviral.

Your codes should enable you to organise your data into manageable ‘bites’ that can be interpreted with ease in the next step. The range of opinions, for example, can be captured during interpretation, so you do not need a code for every viewpoint. However, once coding is completed it is easy to join codes together, but very difficult to divide up a code without re-analysing all the coded text. Codes, therefore, should be close to the data and not too broadly defined. The researcher should give the code a short descriptive label and be clear as to what data belong to this code and what is excluded. This can be defined with examples in the thematic index. Codes should be mutually exclusive,^18^ although the same passage of text can have multiple codes attached.

### Indexing

In this step, the codes in the thematic index are applied systematically to all the qualitative data. Text is annotated with all the codes that are being applied to it, usually by highlighting the passage and noting the code used in the margin.^14^ This is not a mechanical process and often requires the use of judgement to decide on the most appropriate code.

### Charting

In this step, the data are re-arranged into a series of charts that bring all the data with the same code together in one place from all the data sources.^14, 17^ Charts are often constructed using the framework of the thematic index (i.e. one chart per major category) or can be aligned with another conceptual framework that makes sense of the study aim and objectives. Each chart will usually be a matrix in which each column is a particular code and each row a particular data source. The cells of the chart may contain the highlighted quotation or a summary of the point being made. Each cell should be traceable back to the original data source, page and line number. One can use tables in a Word document, cells in an Excel spreadsheet or specially designed software to create charts.

### Interpretation

The researcher will now read each chart and interpret the data. As the charts bring all the data related to a particular category or concept together in one place, it is easy to establish the range and nature of the phenomenon of interest.^16^ Depending on the research aim and objectives, the following interpretations may also be necessary: how respondents defined the characteristics of key concepts, how emerging themes can be organised into a typology (a range of types of cases), or how patterns in the data suggest associations or explanations. In interpreting the data, it may be useful to pay particular attention to ‘deviant cases’ that contradict or negate the main findings.^15^ Contradictory and deviant experiences may be a rich source of information allowing for a further understanding of the phenomenon and should not be discarded just because they are a minority view.

### Confirmation

It can be helpful to confirm your interpretation of the findings with your respondents – so-called ‘respondent validation’.^19^ This is, however, not feasible or even desirable in all studies. Triangulation (see below) of findings with data from different sources and methods can help confirm the validity of the interpretation. Having several researchers independently analyse and confirm their interpretation with each other can also assist.^15^

## Checking for trustworthiness

Qualitative research should be written up with enough clarity regarding the processes that were employed so as to enable the reader to evaluate the scientific rigour of the study, hence enabling acceptance or refutation of the findings.^20^ The concepts and terminology used to describe the trustworthiness of qualitative research findings are different from quantitative research. The criteria for trustworthiness (verification) are credibility (for internal validity), transferability (external validity), dependability (reliability) and confirmability (objectivity).^21^ Table 2 displays the various strategies employed to achieve each criterion.

**TABLE 2:** Criteria for trustworthiness of qualitative research.

Criterion	Strategy employed
Credibility	Prolonged engagement Peer briefing Triangulation Member checks
Transferability	Providing thick description Purposive sampling
Dependability	Create an audit trail Triangulation
Confirmability	Triangulation Practise reflexivity

### Credibility

*Credibility* is concerned with the validity of the conclusions that are drawn from the data and how these conclusions match the reality being reported on. There are many aspects of the study design and reporting that affect the credibility of the work: recognised research methods, sufficient engagement with the phenomenon of interest, checking the transcriptions and emerging themes with the informants (so called ‘member checking’ and ‘respondent validation’), debriefing of the researcher with their mentor or supervisor, peer scrutiny of the process and triangulation.

Triangulation has been described as ‘the process of corroborating evidence from different individuals, types of data, or methods of data collection’.^20^ For example, data sources could be interviews, observations of events in the field (including the informants’ non-verbal expressions), information obtained from semi-structured questionnaires or other relevant documents. Triangulation could also refer to obtaining information from various different types of informants on the same phenomenon.^20, 22^ The effect of triangulation is to render a more holistic picture of the phenomenon under study and to prevent undue reliance on a single data collection method or source.^23^

### Transferability

*Transferability* refers to how well the study conclusions can be applied to other similar settings. The ability of others to judge whether the findings can be transferred depends on a detailed description of the study setting, the selection of participants and the findings. This is often referred to as a ‘thick description’.

### Dependability

*Dependability* refers to the extent to which similar findings would be obtained if the study were repeated.^24^ However, variability should be expected in qualitative studies as the focus is on ‘the range of experience rather than the average experience’.^25^ The best way of supporting the dependability of the research is to ensure that the methods are described in sufficient detail that they could be replicated by someone else (a step-by-step ‘audit trail’) and any limitations are discussed. Triangulation of methods will also improve the dependability of the findings.

### Confirmability

*Confirmability* refers to the degree of objectivity of the researcher in data collection and reporting. The reader wants to ensure that the results are truly based on the data and not the characteristics or preferences of the researcher. Triangulation of the findings with other researchers and methods can help with this, but all qualitative researchers must also account for their own reflexivity.

Reflexivity refers to the researcher's awareness of the self as a research instrument.^26^ The quality of the data obtained through interviewing or observation will, to some extent, depend on the nature of the interviewer. The extent to which they are open, detached, curious and unprejudiced will affect the responses that they receive and/or what they observe. Issues such as power, hierarchy, class, culture and language may also influence the responses.^27^ The researchers should be aware of their own reactions (thoughts, feelings, judgements etc.) and pet theories that may influence how they interpret the data during the analytical process. Qualitative research does not claim to be free of subjectivity, but should strive to be conscious thereof and to describe how the researchers have accounted for the subjectivity as part of the research process.^27^ Describing the researcher's credentials, training, occupation and prior relationship to the subjects of the research helps to demonstrate in the proposal that you are thinking about reflexivity.

## Computer-assisted qualitative data analysis

These enhance the analytical process by making it easier and quicker to code, collate, interpret your data and select quotations for the final report.^28^ The most popular programmes are HyperRESEARCH (ResearchWare, Inc.), NVivo (QSR International) and ATLAS.ti (ATLAS.ti Scientific Software Development GmbH). It should be noted that these programmes do not interpret the data for you, but simply speed up the process and make the handling of large amounts of data both manageable and systematic.^15, 29^

## Presentation of findings

A checklist for explicit and comprehensive reporting of qualitative studies has been suggested by Tong, Sainsburg and Craig in order to promote complete and transparent reporting amongst researchers of interview and focus group studies.^30^ The checklist is based on three domains: research team and reflexivity, study design and reporting on findings. The study design has been described in another article in this series and reflexivity has been described above.

You should start writing up your findings as soon as you have completed the interpretation of your data. It is customary to start the results section of your thesis with an overview or profile of your research subjects or respondents. Sometimes this can be narrative, but a table may also help to clarify who they were along with their key characteristics. It is important to remember to collect this information (e.g. sex, age, qualifications or whatever is relevant to your study) when you interview or observe them.

The presentation of results will depend on the level of analysis. For example, a simple narrative may be presented chronologically or according to key topics, subheadings may be used to present a series of emergent themes or results may be presented to establish a new theory. In primary care research it is common to present a series of themes. In this example, the main theme may be given as a subheading and then the subthemes and main points are described in your own words according to your interpretation of the data. It is important to remember that these are not quantitative data and should not usually be reported in terms of the number of people who expressed a particular viewpoint.^15^ Each main point is usually supported by a quotation from the raw data that speaks to the trustworthiness of your analysis. Quotations also help to connect the reader to the phenomenon that you are describing and to bring it alive.^16^ The voice behind each quotation can be identified in brackets, as long as this does not clash with the need for confidentiality. It is, however, helpful to the reader to know in general terms whose voice the quote represents (e.g. patients or health workers). It is also helpful to number or code the different sources so that the reader can see you have used a wide variety of sources in supporting your findings.

An example of presentation of results from a study by Ifebuzor et al., conducted on the perceptions of parents about the skin conditions of their children in Francistown, Botswana, is given in Box 2.^31^

BOX 2 Example of how to present qualitative results.^31^

Participants did not identify their skin condition in terms of Western medical knowledge and did not view it as serious:

‘I do not know [*what it is*], but every child has it and we do not see it as a problem. I also have it on my body, but it is not causing any problems [*for*] me.’ (TN, Female, 23 years)

‘Yes, I always see it on the bodies of the children, but I do not know anything about it.’ (BM, Female, 77 years)

**FIGURE 1:**
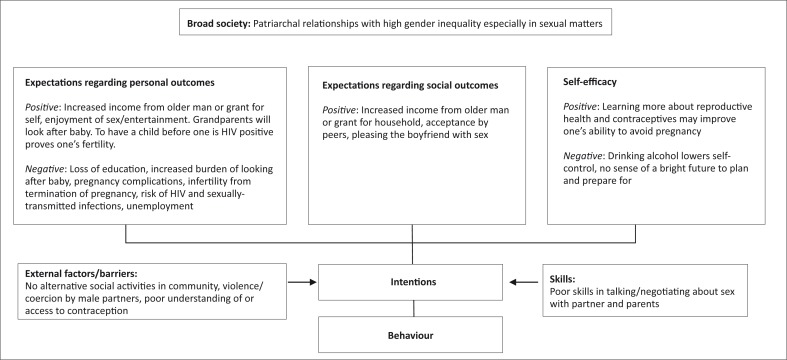
Example of a conceptual framework.

At the end of the results section, it can be helpful to try and summarise the key findings for the reader using a conceptual framework. For example, this could be in the form of a picture, a diagram such as a fishbone (e.g. for factors influencing an adverse event), a table (e.g. a strengths–weaknesses–opportunities–threats [SWOT] matrix) or a theoretical framework taken from the literature (see Figure 1 for an example from a study exploring the factors that influence teenage pregnancy, where social cognitive theory was used for the conceptual framework).^32^

## Conclusion

This article has described the broad range of qualitative methods and types of analysis and then focused on a stepwise approach to content analysis using the framework method as a specific example. It has described the factors involved in determining the trustworthiness of qualitative data analysis and, finally, has outlined how to present your findings in a thesis or scientific article.
